# Computational identification of rare codons of *Escherichia coli *based on codon pairs preference

**DOI:** 10.1186/1471-2105-11-61

**Published:** 2010-01-28

**Authors:** Xianming Wu, Songfeng Wu, Dong Li, Jiyang Zhang, Lin Hou, Jie Ma, Wanlin Liu, Daming Ren, Yunping Zhu, Fuchu He

**Affiliations:** 1 School of Biological Science and Technology, Shenyang Agricultural University, Shenyang 110161, PR China; 2 State Key Laboratory of Proteomics, Beijing Proteome Research Center, Beijing Institute of Radiation Medicine, Beijing 102206, PR China

## Abstract

**Background:**

Codon bias is believed to play an important role in the control of gene expression. In *Escherichia coli*, some rare codons, which can limit the expression level of exogenous protein, have been defined by gene engineering operations. Previous studies have confirmed the existence of codon pair's preference in many genomes, but the underlying cause of this bias has not been well established. Here we focus on the patterns of rarely-used synonymous codons. A novel method was introduced to identify the rare codons merely by codon pair bias in *Escherichia coli*.

**Results:**

In *Escherichia coli*, we defined the "rare codon pairs" by calculating the frequency of occurrence of all codon pairs in coding sequences. Rare codons which are disliked in genes could make great contributions to forming rare codon pairs. Meanwhile our investigation showed that many of these rare codon pairs contain termination codons and the recognized sites of restriction enzymes. Furthermore, a new index (F_rare_) was developed. Through comparison with the classical indices we found a significant negative correlation between F_rare _and the indices which depend on reference datasets.

**Conclusions:**

Our approach suggests that we can identify rare codons by studying the context in which a codon lies. Also, the frequency of rare codons (F_rare_) could be a useful index of codon bias regardless of the lack of expression abundance information.

## Background

Codon usage bias has attracted attention for several decades. Since the 1970s, the unequal use of synonymous codons has been confirmed in many organisms. To date, the codon usage patterns in many organisms have been interpreted for diverse reasons. For instance, there have been some different influence factors proposed by researchers: the abundance of isoacceptor tRNA[1,2], amino acid composition[3], mRNA secondary structure[4], the efficiency of translation initiation[5], GC content[6], gene length[7,8], protein structure[9] and so on. Although there is still no final verdict on the formation mechanism, codon bias has been widely used to estimate and compare the expression level of endogenous genes, change the efficiency of expression of exogenous genes[10-12], identify horizontal transfer genes from other organism[13], judge the relationship of evolution[14], and confirm the coding sequences.

From early investigations in *Escherichia coli*[1], it was found that usage of preferred codons in genes was positively correlated with their respective major isoacceptor tRNA levels, and this was explained as an adaptation of highly expressed genes to translational efficiency. Since then, extensive studies on codon usage bias have been performed in other organisms such as *S. cerevisiae*[1,15,16], *Drosophila*[17] and *C. elegans*[18]; and the results of this research have supported the dominant theory above. One long accepted principle of this theory is that highly expressed gene must show high codon usage bias. However, with the development of high throughput technology for gene sequencing and expression level detection, doubts over this theory have increased gradually[1,19-26].

In order to describe and measure the degree of codon bias, a series of indices have been developed and applied to codon bias analysis over the past thirty years. A survey of the literature indicated that in some prokaryotes, many indices exhibit a positive correlation with the gene expression level, such as CAI (Codon Adaptation Index)[27], CBI (Codon Bias Index)[28], and Fop (Frequency of optimal Codons)[1]. However, in some eukaryotes, especially for higher eukaryotes, the correlation between codon bias and expression level is extremely weak[1,29]. Therefore the balance between translational selection and mutational bias has been used to account for the codon bias observed in these organisms. These paradoxical results remind us there must be a more complicated mechanism for forming codon bias in different species beyond what this correlation suggests.

In recent years, codon pair preference has also become a popular topic in the field of codon bias when attention is turned to the context in which a codon lies. The existence of codon pair preference has been confirmed by many investigations in several organisms[30-36]. The exploration of the mechanism for driving the formation of codon bias attracts more attention on the level of protein. For example, whether translation optimization might be a primary selective pressure or translation apparatus could inflict selective pressure[36]. Seldom researchers care about the level of DNA or mRNA.

*Escherichia coli *are frequently used as host cells in the study of expressing exogenous proteins efficiently. According to previous gene engineering operations, many genes of eukaryotes can not be expressed smoothly in *Escherichia coli*; and one effective method to improve the expression level or to avoid the frame shift mutation is to replace the usage of "rare codons" with synonymous codons. To search for clues for explanation, the stability of genes, on the level of DNA or mRNA, should be taken into consideration so as to play a role in determining gene expression [37-43].

In this paper, we introduced a novel computational method to identify rare codons of *Escherichia coli*. Moreover, as a new index based on this method, F_rare _was developed to measure codon bias objectively.

## Results

### The analysis of codon usage pattern by CodonW

From the analysis of genes in *Escherichia coli *by CodonW, we derived the codon usage patterns and the ranked results of synonymous codons (Additional file 1). In addition, extreme similarity between essential genes (see method section)[44-46] and whole genes in *Escherichia coli *was found, which implies that there should be a basic "rule" for codon usage patterns in *Escherichia coli *(table 1). If expression efficiency on the protein level cannot serve effectively as the "rule"[1,19-26], perhaps further research should focus on the contribution of selective pressure to the stability of genes.

**Table 1:** Nonparametric correlation analysis (Spearman's rank correlation) of codon usage patterns between essential genes and whole genes in *Escherichia coli*

		Whole_ecoli	Ecoli234_essential
Whole_ecoli	Correlation Coefficient	1.000	.845**
	Sig.(2-tailed)	.	.000
	N	64	64
Ecoli234_essential	Correlation Coefficient	.845**	1.000
	Sig.(2-tailed)	.000	.
	N	64	64

**. Correlation is significant at the 0.01 level (2-tailed)

### Identification of rare codons of *Escherichia coli *based on codon pairs preference

1. We calculated the occurrence frequency of each kind of different six-nucleotide strings in 4289 sequences of *Escherichia coli *k12 in two ways (see method section). Using the criterion derived from statistical analysis, the "rare codon pairs" and "normal codon pairs" were defined. As a result, we obtained 1160 "rare codon pairs" and 2890 "normal codon pairs" (Additional file 1).

2. Thirteen rare codons (GGA, CTC, TAG, CTA, ACA, GAC, AGG, AGA, CCC, GGG, GAG, ACT, and ATA) were identified by the statistical test method for hypergeometric distribution, which was used to evaluate the contribution of the sixty-four codons to the rare codon pairs(Additional file 1). It is exciting to find that these "rare codons" were the very codons which have been regarded as limiting factors of exogenous gene expression by experimental verification over a long period of time[44,47-58].

### F_rare _(the frequency of rare codons) was developed as a novel index of codon bias

1. We can calculate the F_rare _value of genes based on the rare codons identified by the method mentioned above. The strong inverse correlation between F_rare _and CAI suggests that experiments for deriving expression information are dispensable for quantification of codon bias.

2. Using the rank sum test(table 2), it was noted that the F_rare _values of essential genes were lower than those of nonessential genes. We can thus conclude that the essential genes avoid the use of rare codons because the essential genes are indispensable for sustaining cellular life.

**Table 2:** Mann-Whitney Test

Information of sum of ranks				Result of Mann-Whitney Test: Test Statistics ^a^	
group	N	Mean Rank	Sum of Ranks	Mann-Whitney U	F_rare__ess_nonessential 2374.50
1	234	127.65	29869.50	Wilcoxon W	29869.50
2	4055	2261.41	9170035.50	Z	-25.630
Total	4289			Asymp.Sig. (2-tailed)	.000

a. Grouping Variable: group

### Exploring the factors related to stability of genes in rare codon pairs

1. Some relationship between the rare codon pairs and stability of gene could be found from the references and database (The Restriction Enzyme Database http://rebase.neb.com/rebase/rebase.html) searching. There are nonsense codons (TAA, TAG, TGA) and recognition sites of restriction enzymes or methylases in some rare codon pairs. For instance, many of the rare codon pairs in the "rare group" are involved in affecting the stability of gene (Additional file 1: 94 rare six-nucleotide strings have been found containing recognition sites of restriction enzymes and 202 rare six-nucleotide strings have been found containing nonsense codon. Moreover, the investigation will continue).

2. We noticed that there are also some rare six-nucleotide strings present in the "normal groups". The common characteristic of these strings is that they contain "nonsense codons" (TAG, TGA, TAA) which is proved to be an important element of mRNA's instability[43]. Additionally, we have found the appearance of "TAG" in rare codon list and it can support that "TAG" is the most inefficient stop codon in *Escherichia coli*.

## Discussion

Compared with the results of experiments, we suggest that it is feasible to identify rare codons of *Escherichia coli *based on codon pair's preference. From the identification consequence, not all seldom used synonymous codons are "rare codons" that can limit the expression level of heterologous genes.

In recent years, there has been some debate over the significant difference of the codon usage patterns existing in different kinds of genes in the same species[44,59,60]. In our study, the codon usage patterns of essential genes were selected to compare with those of the whole genes in *Escherichia coli*. These essential genes are very important for maintenance of the basal cellular function, so they are likely to be common for all cells and not be horizontal transfer genes from other organism. Therefore, the extreme similarity of codon usage patterns between essential genes and whole genes in *Escherichia coli*, would suggest the existence of a common rule that can control the pattern.

The expression level may be affected and controlled by many factors. Thus it seems unimaginable that the expression level in dynamic change could control the codon usage pattern. Furthermore, to our knowledge, the abundance of isoacceptor tRNA, which could be a powerful evidence to support the classical theory of Ikemura[1], cannot be precisely measured until now. Instead, we focused on the relationship between codon usage and gene stability. This relationship is important for the connection between gene and protein in the translation control system of *Escherichia coli*.

In the study of codon bias, CAI[27,61-64], CBI [65]and Fop[1] were commonly used for analysis. Although these indices have been revised [21] several times, it is still necessary for researchers to obtain a reference dataset containing gene expression abundance data in calculating them. In addition, though the high correlation between gene expression abundance and codon bias index has been found in prokaryotes[1,19,66] and some eukaryotes[17,67], there isn't enough evidence to support it in *Homo sapiens*[3] and other eukaryotes. F_rare _value, which didn't depend on the reference dataset, was developed in this study to compare and scale the codon usage pattern basis on the identified rare codons. The strong inverse correlation between F_rare _and CAI indicates that the usage of rare codons affects the process and consequence of translation.

The essential genes are expected to be stable because of their function as foundations of life. Thereby essential genes dislike rare codons and possess lower F_rare _values. As we know, many genes of *Homo sapiens *introduced in *Escherichia coli *directly cannot express well. We argue that this might be the result of mass occurrence of rare codons of *Escherichia coli*, which can induce instability to these genes. From this, we foresee that in order to improve the expression level in the operation of heterologous gene expression, we should modify and replace the identified rare codons to avoid the appearance of rare strings. We could also estimate the stability of exogenous genes by calculating the F_rare _value using the rare codons of the host.

## Conclusions

We introduced a novel computational method to identify rare codons in *Escherichia coli *based upon the codon pair's preference. By comparing the thirteen identified rare codons with the results of published experiments, we have proved that our method would be helpful to the study of heterologous gene expression operation. For description of the codon usage pattern by considering the rare codons, F_rare _was developed as a new index without requiring expression level information.

## Methods

### Gene sequences of *Escherichia coli*

1. 4289 gene sequences of *Escherichia coli *K12-MG1655

Gene sequences of *Escherichia coli *K12-MG1655 were downloaded from http://cmr.tigr.org/tigr-scripts/CMR/shared/MakeFrontPages.cgi?page=batchdownload.

2. 234 essential gene sequences *Escherichia coli*

Essential genes are genes that are indispensable to support cellular life. These genes constitute a minimal gene set required for a living cell. The functions encoded by essential genes are considered a foundation of life and therefore are likely to be common to all cells[45,46].

Information of essential genes in *Escherichia coli *were download from PEC (The Profiling of Escherichia coli chromosome) database http://www.shigen.nig.ac.jp/ecoli/pec/index.jsp, then a Perl program was made to obtain the sequences of essential genes from the 4289 whole gene sequences. At last, 234 essential gene sequences were obtained.

### Tools for analysis

#### Perl programs

A series of programs were written using the Perl language(ActiveState Perl, v5.8.4) and run in DOS. These programs were used to obtain the gene sequences and to do statistical analysis after completing search processes of the codon pairs. All programs written as part of this study are freely available on request from the author.

#### CodonW

The analysis of codon usage patterns were performed using the software CodonW (downloaded from http://sourceforge.net/project/showfiles.php?group_id=129506&package_id=141931&release_id=307994.)

#### BioEdit

All the gene sequences were loaded into BioEdit (Version 7.0.0) and translated into protein sequences respectively according to the general codon table.

#### Other tools

The output files generated by CodonW and some Perl programs were loaded into Excel (Microsoft) for display and further analysis. Also, some statistical tests were done using SPSS(SPSS 13.0 for windows) and MATLAB (Version 7.0.0.19920) to determine the significance of the analysis results.

#### Analysis of codon usage patterns

To obtain the patterns of codon usage, the files containing fasta sequences of *Escherichia coli *were loaded into CodonW (Additional file 1). In *Escherichia coli*, the codon usage pattern of essential genes was compared with that of whole genes by Spearman's rank correlation analysis. It was noteworthy that the codon usage patterns of essential genes and whole genes are uniform as shown by Nonparametric Correlations (table 1).

### Identification of rare codons based on codon pairs preference

#### Codon pairs searching and statistical test

1. There are a total of 4096 (64 × 64 = 4096) different six-nucleotide strings made up of 2 codons. An array containing these strings was made for search in gene sequences of *Escherichia coli*.

2. Several Perl programs were made to search the 4096 strings in 4289 sequences of *Escherichia coli *k12; then, the "rare strings" and "normal strings" were defined by statistical analysis.

1) Searching the strings in accordance with open reading frame

One string made up of two amino acids would correspond to several six-nucleotide strings according to synonymous codon's encoding rule. So we first calculated the frequencies of all the six-nucleotide strings and the corresponding two amino acid strings, by open reading frame, within all gene sequences and protein sequences respectively in *Escherichia coli*. Then the statistical test method for binomial cumulative distribution was implemented to get a P_1 _value of each codon pair, by which we can display the probability of the real frequency if we assumed that codon usage was random (we got P1 values of 4050 strings after excluding the strings which are not adapted to analysis because they only contain "ATG" or "TGG" or their corresponding two amino acids strings don't exist in protein sequences).

*p*: the probability of a codon pair's occurrence corresponding to any given two amino acids string based on encoding rule. 

k: the frequency of a codon pair according to open reading frame;

m: 0 ≦ m ≧ k;

n: the frequency of the corresponding 2 amino acid string encoded by the codon pair in protein sequence;

syn(i): the degeneracy of the amino acid coded by i.

2) Searching the strings in spite of open reading frame

The actual occurrence frequency of all the six-nucleotide strings in gene sequences was gotten by general search in spite of open reading frame, and also P_2 _was calculated by the same method for binomial cumulative distribution above.

*p*: the probability of a six-nucleotide string's occurrence because of possible composition of four types of nucleotides. 

k: the frequency of a six-nucleotide strings by general searching in 4289 sequences;

m: 0 ≦ m ≧ k;

n: (N_i _is the number of nucleotides in gene i).

3) The criterion for dividing all codon pairs into "rare" and "normal" groups

Through the analysis above, we got two P values (P_1_, P_2_) for each codon pair, then a cutoff value P_0 _(P_0 _= 0.01/4096/4289 = 5.69225 × 10^-10^) was made to be the criterion. For a codon pair, If P_1 _and P_2 _are both less than P_0 _(P_1 _< P_0 _and P_2 _< P_0_), it will be defined as "rare codon pair". Otherwise, it will be thrown into the "normal" group. As a result, we obtained 1160 "rare codon pairs" and 2890 "normal codon pairs" (Additional file 1).

3. Lastly, the statistical test method for hypergeometric distribution was used by MATLAB to find out how the sixty-four codons contribute to the rare strings. After the codons were ranked by P_hyp _value of this test, we realized that some rare codons in the front rank could make great contributions to rare strings; so we identified them as "rare codons" (Additional file 1).

x: the frequency of a codon in "rare group"

N: N = 4 × N_1 _(N_1_: the number of codon pairs in "rare group"; N_2_: the number of codon pairs in "normal group")

M: M = 4 × N_1_+4 × N_2 _(N_1 _and N_2 _have been multiplied by 4 because a six-nucleotide string will contain 4 triplets starting from different point when open reading frame is ill-defined)

K: the frequency of a codon in "normal group"

### A new index "F_rare_" (frequency of rare codon) is helpful to describe the codon usage pattern

Just as "Fop"[1] has been used to predict highly expressed genes, F_rare _(the frequency of rare codon) value was introduced to show the codon usage pattern from a different standpoint. Here we defined F_rare _of gene g as:

n_i_(g): the count of the codon i in the gene g;

N: the total number of codons in gene g;

syn(i): the degeneracy of the amino acid encoded by i

In the expressions above, the sum is taken over all the rare codons. Through corresponding analysis we found that there is strong negative correlation between F_rare _and CAI (or CBI/Fop) in *Escherichia coli *(table 3).

**Table 3:** Correlation analysis between F_rare _value and classical codon bias indices

		F_rare__value	CAI	CBI	Fop
F_rare__value	Correlation Coefficient	1.000	-.729**	-.640**	-.644**
	Sig.(2-tailed)	.	.000	.000	.000
	N	4289	4289	4289	4289
CAI	Correlation Coefficient	-.729**	1.000	.905**	.933**
	Sig.(2-tailed)	.000	.	.000	.000
	N	4289	4289	4289	4289
CBI	Correlation Coefficient	-.640**	.905**	1.000	.989**
	Sig.(2-tailed)	.000	.000	.	.000
	N	4289	4289	4289	4289
Fop	Correlation Coefficient	-.644**	.933**	.989**	1.000
	Sig.(2-tailed)	.000	.000	.000	.
	N	4289	4289	4289	4289

**. Correlation is significant at the 0.01 level (2-tailed)

**Figure 1:**
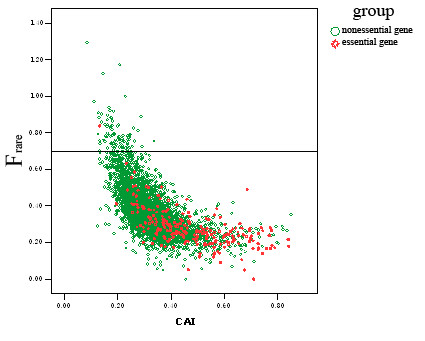
**Comparison of F_rare _values between essential genes and nonessential genes by scattering points diagram**.

We found that the F_rare _values of essential genes tend to be less than those of nonessential genes (figure 1), which means essential genes prefer to reject the "rare codon" over other genes. Also the rank sum test (Mann-Whitney Test) was applied to determine whether a significant division of "essential genes" and "nonessential genes" by F_rare _values exhibited (table 2). As a result, the P values of rank sum test are small enough to make sense of the division.

## Authors' contributions

SW, DLi and JZ participated in developing programs. LH, JM, WL performed the statistical analysis. XW conceived of the study, and participated in its design and coordination and drafted the manuscript. DR, YZ, FH participated in the design of the study and helped to draft the manuscript. All authors read and approved the final manuscript.

## Supplementary Material

Additional file 1 **The important dataset of this study has been saved in an excel file**. Sheet 1a: codon usage patterns. Sheet 2a:1160 rare codon pairs. Sheet 2b: 2890 normal codon pairs. Sheet 3a: identification of rare codons. Sheet 4a: 94 codon pairs containing recognition site. Sheet 4b: 202 codon pairs containing nonsense codon. Click here for file
